# Remittance flows and US monetary policy

**DOI:** 10.1007/s10290-022-00478-x

**Published:** 2022-09-24

**Authors:** Immaculate Machasio, Peter Tillmann

**Affiliations:** 1 The World Bank Group, Washington, USA; 2 Justus Liebig University Giessen, Giessen, Germany

**Keywords:** Remittances, Migration, Business cycle, Monetary policy, Spillovers, F24, F41, E52, O11

## Abstract

Remittance inflows are driven by macroeconomic conditions in the home and the host economies, respectively. In this paper, we study the effect of US monetary policy on remittance flows into economies in Latin American and the Caribbean. The role of Fed policy for remittances has not yet been studied. We estimate a series of panel local projections for remittance inflows into eight countries. A surprise change in US monetary conditions has a strong and highly significant negative effect on inflows. Our finding remains robust if we change the sample period or include additional variables. Hence, our paper establishes a remittance-channel through which the Fed affects the business cycle abroad.

## Introduction

The inflow of remittances is an important source of financing for many emerging market economies. Remittance flows are considered more stable than other forms of private capital flows.^1^ Nevertheless, remittances exhibit a cyclical pattern. Migrant workers respond to macroeconomic conditions in both their home as well as their host economy.^2^ One key driver of the business cycle in the host economy is monetary policy. Hence, monetary policy shocks should be a source of fluctuations of remittance flows. A large literature shows that if the Fed tightens its policy stance, income falls and unemployment increases. Both variables should also drive migrant workers’ ability to transfer money to their families at home. A monetary tightening of the Fed could lower the inflows of remittances, thus contributing to business cycle volatility in developing economies. We refer to this as the remittance-channel of cross-border monetary policy transmission.

Against this backdrop, this paper studies the effect of US monetary policy on remittance inflows into economies in Latin America and the Caribbean. We concentrate on Latin America and the Caribbean because for our eight sample countries, the majority of oversees workers reside in the US, such that we can clearly identify the U.S. as the host economy. Since the U.S. is the main source of flows, we can treat inflows into, say, Mexico, as U.S. outflows. We use quarterly data on remittance inflows between 2000 and 2020 into Mexico, Guatemala, El Salvador, Costa Rica, Panama, Ecuador, Jamaica and Colombia. A second criterion for the selection of countries is the availability of quarterly remittance data.

We estimate a series of panel local projection models. This class of models is used widely in order to quantify the impact of US monetary policy and other shocks on business cycle variables. Hence, we draw on the established literature and investigate the sensitivity of remittance inflows to unexpected changes in US monetary conditions.

As a key contribution, we find that remittance inflows are highly sensitive to monetary policy shocks. An unexpected tightening of Fed policy reduces the inflow of remittances. A policy surprise one standard deviation in size reduces the inflow of remittances by about 0.8%. Hence, monetary policy affects foreign business cycles through its effect on remittance flows.

This result survives when we end the sample in 2019, i.e. before the Great Lockdown imposed to fight the COVID-19 pandemic, or when we use an alternative definition of the dependent variable. We also show that the results remain unchanged if we control for natural disasters in migrants’ home countries. The impact of monetary policy is also symmetric with respect to tightening and easing shocks. We find that the response of remittance flows is similar in terms of magnitude to the response of inflows of foreign direct investment 4FDI), which is the second most important type of private capital inflows into our sample countries besides the inflow of remittances. Finally, we shed light on the role of the real exchange rate. A US policy tightening leads to a real depreciation of the dollar, which should ceteris paribus raise remittance inflows, as migrant workers need to sacrifice fewer US goods in order to transfer resources to their families in the home countries. Controlling for the response of the real exchange rate, however, leaves our key results unaffected. Hence, we establish firm evidence for an economically relevant remittance-channel of monetary policy.

The remainder of this paper proceeds as follows: in Sect. 2, we survey the related literature. Section 3 provides stylized facts on remittance flows for our sample countries. The empirical model is introduced in Sect. 4, while Sect. 5 discusses the results. Section 6 focuses on the role of the real exchange rate response for remittance flows and Sect. 7 concludes.

## Related literature

Our study is related to the huge body of literature on the cyclical properties of remittance flows. Countercyclical remittances potentially facilitate the process of smoothing economic fluctuations while procyclical flows are likely to amplify cycles. Theory suggests that migrants’ motives to remit shape the behavior pattern of remittances over cycles because altruism results into countercyclical remittances while self-interest leads to procyclical flows (Lucas and Stark, 1985).

In examining business cycle effects of immigration to the US using panel data on Mexican workers in U.S. states between 2011 and 2014, Mendoza and Ashby (2019) provide evidence to suggest that immigrants are attracted by stronger performance of the U.S. economy while a booming economy in source countries tends to decrease emigration. They also find that increased remittances to Mexico is associated with reduction in outward migration from Mexico. Other studies attribute remittance inflows to consumption smoothing. Mandelman and Zlate (2012) demonstrate this using a model, which captures business cycle effects in the United States and Mexico. They suggest that remittance inflows to Mexico serve as an insurance mechanism and enhance consumption smoothing.

Studies that shed light on the cyclical nature of remittances with respect to domestic macroeconomic variables typically provide mixed results. Frankel (2011) expounds on the smoothing hypothesis and suggests that remittances are procyclical in nature when income in a migrant’s destination country is taken into consideration and countercyclical with respect to income in migrants’ home country. In contrast, Lueth and Ruiz-Arranz (2008) find that remittances fall when exports dwindle and GDP growth slows, a feature highlighting the procylicality of remittances. Their findings suggest that altruism is not necessarily the prime reason for remittances since remittances do not seem to respond to natural disasters in migrants’ home countries.

Research results on determining whether remittances reduce the probability of current account reversals by Bugamelli and Paterno (2011) also support the notion of countercyclical properties of remittance inflows. In the same vein, De et al. (2019) evaluate remittance behavior over the business cycle. Their findings show that remittances exhibit stability during episodes of high volatility in business cycles, thus suggesting that remittances can facilitate the process of consumption smoothing during economic hardships.

Other authors find more mixed results (Durdu and Sayan, 2010; Mughal and Ahmed, 2014; Vargas-Silva, 2008).

A recent study by De et al., 2019 shows that remittances are predominantly acyclical. Their research also suggests that remittances are more resilient and less volatile than other types of inflows such as FDI and Official Development Assistance (ODA). Buch and Kuckulenz (2010) show that remittances respond less to macroeconomic conditions than other types of private capital inflows.

In explaining the procyclical and countercyclical nature of remittances, Machasio and Tillmann (2017) distinguish between North–South and South-South flows and show that an increased share of remittances from low-income countries significantly affects the cyclical nature of aggregate inflows.

There is no systematic evidence yet on the responsiveness of remittances to U.S. monetary policy. Hence, we contribute to this literature by studying a specific channel through which the business cycle conditions in the host economy affect worker remittances. By doing so, we also add to the literature on cross-border spillovers of US monetary policy (Ahmed et al., 2021; Dahlhaus and Vasishtha, 2020; Tillmann, 2016).

## Descriptive statistics

Our findings stem from a series of local projections for remittance flows from the U.S. into eight Latin American and the Caribbean economies. The United States is the top destination country for immigrants worldwide, with about 51 million international migrants residing within US borders in 2019 (UNDESA, 2019). This accounts for about 19 percent of the total worldwide international migrant stock, which was about 272 million in 2019. Top 10 migrant source countries include Mexico, China, India, the Philippines, Puerto Rico, Vietnam, El Salvador, Cuba, the Republic of Korea and the Dominican Republic. The U.S. is the largest remittance source country with an estimated remittance outflow of $178 billion in 2019.

This study uses quarterly observations available for all variables that cover the period from 2000Q1 to 2020Q4 for Mexico, Guatemala, El Salvador, Costa Rica and Panama, Ecuador, Jamaica and Colombia. The selection of sample countries in our study is dictated by the availability of quarterly remittances data. Moreover, we select the eight countries situated in Latin America and the Caribbean because a considerably high percentage of migrants originating from these countries reside in the U.S. Quarterly remittances flow data for Colombia, Ecuador and Jamaica is retrieved from the respective central bank websites and the remaining remittance data is taken from the IMF BOP statistics.

Table 1 depicts a number of descriptive observations for our sample countries. In particular, we sketch the recipient countries’ migration patterns with respect to the US. We use bilateral remittances data provided by the World Bank-KNOMAD and bilateral migration matrix data drawn from UNDESA to compute the share of remittances emanating from the US as well as the percentage of migrants residing in the US. The table also reports per capita income obtained from the WDI. To measure the income gap between the US and recipient countries, we subtract the GDP per capita value of the US from an individual country’s GDP per capita.

**Table 1:** Some facts about our sample countries

Country	Number of emigrants worldwide (2019)	Share of migrant stock in the U.S. (in %)	Share of remittance inflows from the U.S. (in %)	GDP per capita (current USD, 2019)	Income gap per capita (current USD, 2019)	Remittances as a share of GDP, 2019 (in %)
Mexico	11,796,178	97	98	9946	− 55,334	3.07
Guatemala	1,205,644	89	91	4639	− 60,641	13.84
El Salvador	1,600,739	89	91	4168	− 61,112	21.05
Costa Rica	150,400	66	69	12,670	− 52,610	0.86
Panama	161,107	78	79	15,728	− 49,552	0.87
Ecuador	1,183,685	44	47	6223	− 59,057	3.00
Jamaica	1,111,021	68	73	5370	− 59,910	16.19
Colombia	2,869,032	28	37	6425	− 58,855	2.20

*Notes*: The data is taken from World Bank-KNOMAD, UNDESA and WDI

As of 2019, the emigrants residing in the US and originating from the eight countries in our sample accounted for more than 65 percent of total migrants. The high proportion of migrant stocks coupled with huge income gaps between the US and the recipient countries explains the immense volume of remittance inflows received by the sample countries.

**Fig. 1:**
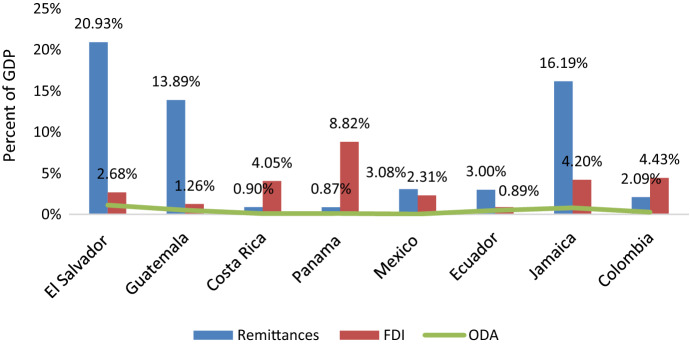
Remittance inflows and other sources of inflows in 2019. *Notes*: The data is taken from World Bank-WDI

Figure 1 shows that in 2019, remittance inflows topped official development assistance in most of the countries in the sample, ranked second to FDI in Costa Rica, Colombia and Panama and accounted for the highest percentage of GDP in El Salvador, Guatemala, Mexico, Ecuador and Jamaica. This underscores the importance of remittance inflows in the recipient economies situated in the Latin America and the Caribbean region.

## The empirical model

We estimate a series of panel local projections following the work of Jordà (2005) and Jordà et al. (2020). Recently, local projections have become a standard tool to estimate the dynamic causal impact of shocks on economic time series. In our application, the time series dimension is relatively short. We use quarterly data between 2000Q1 and 2020Q4. Due to fact that we have only 84 observations per country, we also exploit the cross-country dimensions and estimate the local projections for a panel of eight economies.

Our key variable of interest is the inflow of remittances in quarter *t* to country *i*, ${y}_{i,t}$, with *i* = 1, …, *N*. In our case, we include *N* = *8* countries. We are particularly interested in the response of inflows *h* quarters after a monetary policy shock occurring in *t*. Thus, the dependent variable is ${y}_{i,t+h}$, which is regressed on the US monetary policy shock ${\varepsilon }_{t}^{mon}$

1 $$y_{i,t + h} = \alpha_{i,h} + \beta_{h} \varepsilon_{t}^{mon} + \gamma_{h} {\varvec{X}}_{i,t} + \delta_{i,h} t + u_{i,t + h}$$

The estimate of $\beta_{h}$ gives us the response of inflows *h* periods after the shock. As we expect a monetary policy tightening to reduce remittance inflows, our prior is $\beta_{h} < 0$. Importantly, we impose the restriction that the slope coefficient $\beta_{h}$ is identical across the sample countries. In this regression model, ${\alpha }_{i,h}$ is a county fixed-effect and ${\delta }_{i,h}$ is the country-specific coefficient on the time trend. The vector ${{\varvec{X}}}_{i,t}$ collects a number of control variables, which enter with a coefficient vector ${\gamma }_{h}$, which we also assume to be equal across countries in order to reduce the number of parameters to be estimated. The error term is given by ${u}_{i,t+h}$. The local projections are estimated with least squares. Since the errors will not just be correlated over time, but also in the cross-section, we calculate standard errors following Driscoll and Kraay (1998).

**Fig. 2:**
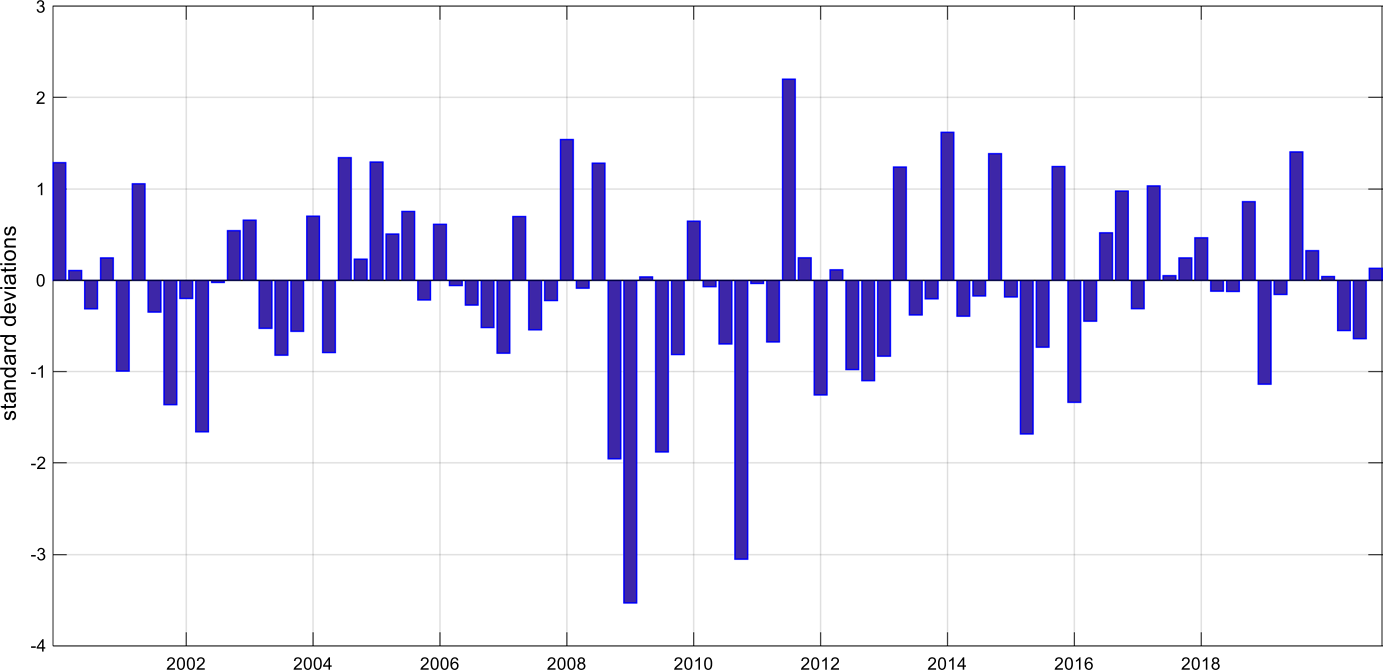
Series of US monetary policy shock. *Notes*: The graph shows the U.S. monetary policy shock provided by Bu et al. (2021). A positive shock corresponds to a surprise tightening of monetary conditions. We cumulated the original shock series to quarterly frequency

The series of monetary policy shocks driving the endogenous variable reflects unexpected changes in US monetary conditions. It should summarize monetary policy changes, which are orthogonal to financial and business cycle conditions. Hence, we need to use a series that is properly identified. We use the monetary policy shock provided by Bu et al. (2021).^3^ These authors identify a US monetary policy shock that equally well reflects conventional policy changes, i.e. interest rate adjustments, and unconventional interventions, i.e. Quantitative Easing and Forward Guidance, such that the zero lower bound on nominal interest is not a problem for our estimation. Moreover, the authors derive a policy shock that is purged from central bank information shocks. Such shocks can occur if central bank actions reveal an informational advantage of the Fed about the state of the economy and would otherwise impair the interpretation of our findings. We cumulate the shock series from Bu et al. (2021) to quarterly frequency and standardize the series such that it is scaled in standard deviations. Figure 2 plots the standardized quarterly shock series that is used for estimating the local projections.

The dependent variable ${y}_{i,t},$ is expressed as the log of seasonally adjusted remittance inflows to selected countries in Latin America and the Caribbean minus the log of the US Consumer Price Index (CPI). Hence, we deflate inflows with the US price level. A fall in the dependent variable following a restrictive monetary policy shock implies that remittance flows fall in real terms. Hence, migrant workers cut their remittance transfers. In an alternative specification, we use the log of remittance inflows in current US dollar. The data source for remittance inflows is the IMF BOP statistics, World Bank-KNOMAD database and the websites of the respective central banks.

Our model includes the contemporaneous realization and six lags of the following control variables: first, log real GDP in the recipient country. This variable is taken from the World Bank and interpolated to quarterly frequency. Lower income in the recipient country should motivate migrant workers to increase their transfers. Second, log US disposable income. An increase in disposable income should lead to higher remittances. Third, the unemployment rate and the log level of employment for the Hispanic part of the US workforce. A higher level of employment and a lower Hispanic unemployment rate should raise remittance inflows into Latin American and the Caribbean economies.^4^ We draw these U.S. data series from the FRED database at the Federal Reserve Bank of St. Louis. Below, we show alternative specifications in which we modify the set of control variables.

The data covers a period from 2000Q1 to 2020Q4 and thus includes the COVID-19 recession. Below, we show how the observations from 2020 affect our findings. We include the countries introduced before, i.e. Colombia, Costa Rica, Ecuador, El Salvador, Guatemala, Jamaica, Mexico and Panama.

## Results

**Fig. 3:**
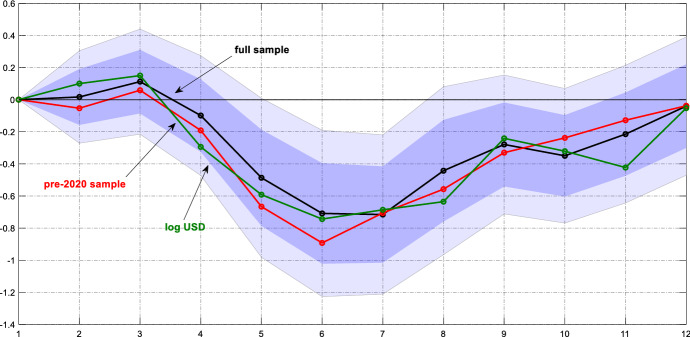
Response of remittance inflows to a US monetary policy shock. *Notes*: The graph shows the estimated $\beta_{h}$ for the baseline model (black line), the baseline model estimated over the 2000–2019 sample (red line) and the model with the dependent variable expressed in current US dollar (green line). The dark (light) blue areas reflect 68% and 90% confidence intervals around the baseline estimates obtained from Driscoll and Kraay (1998) standard errors

Figure 3 presents the resulting impulse response functions following a monetary policy tightening of the Federal Reserve, i.e. the estimated slope coefficient as a function of the horizon *h*. Since the model is symmetric, we can obtain the responses to a monetary easing by flipping the impulse response functions. We show the point estimate and confidence bands that cover 68% and 90% of the estimates.

The figure contains our key result: the inflow of remittances falls significantly after the monetary tightening. A surprise policy tightening of one standard deviation causes a drop in remittance inflows of 0.8% after six quarters. This effect is highly statistically significant. Furthermore, the effect is quantitatively relevant. Given the large volume of remittance flows and their role for business cycles in recipient countries, even a significant but small drop in flows causes strong macroeconomic adjustments. The response is relatively persistent with flows returning to their mean after about 10 quarters. The delay in the peak response does not come as a surprise: the transmission of monetary policy shocks to the real economy needs between four and eight quarters. Workers’ income levels and job perspectives do not deteriorate immediately after the shock, such that the response of remittances is also delayed.

The sample covers the Covid-19 pandemic since February 2020 and the subsequent Great Lockdown, i.e. the deliberate reduction in economic activity in the US and most other countries. In order to find out whether this period of extreme fluctuations affects our findings, we estimate the model over a shorter sample that ends in 2019Q4.^5^ The resulting point estimates are also shown in Fig. 3. Compared to the baseline results, the estimates remain almost unaffected by the reduction in the sample size. Hence, our findings are robust with respect to the disruptions in 2020.

Our dependent variable is deflated by the US consumer price index. The US price level should itself respond to the monetary tightening, which could translate into an adjustment of the deflated series of remittances. We now estimate the model with the (log) level of inflows in current US dollar as the dependent variable, that is, we skip the denominator. The resulting estimates are shown as a green line in Fig. 3. Again, the response of remittances is almost indistinguishable from our baseline estimates, which suggests that deflating the series is an innocuous transformation. Below, we will also show results for remittance flows scaled by nominal GDP in US dollar.

**Fig. 4:**
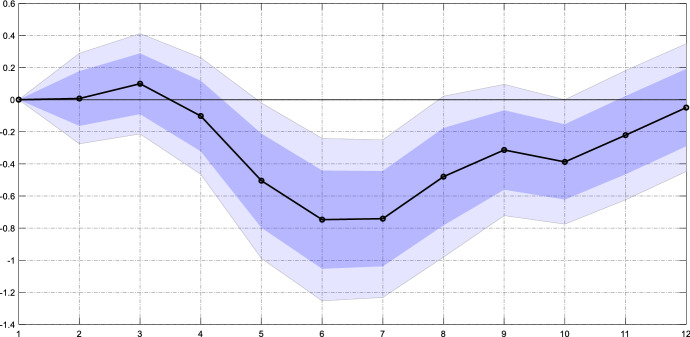
Response of remittance inflows to a U.S. monetary policy shock when controlling for natural disasters. *Notes*: The graph shows the estimated ${\beta }_{h}$ for the baseline model augmented by a series of natural disasters as an additional control variable (black line). The dark (light) blue areas reflect 68% and 90% confidence intervals obtained from Driscoll and Kraay (1998) standard errors

Countries in Latin America and the Caribbean are frequently plagued by natural disasters ranging from hurricanes to earthquakes. A natural disaster that affects people’s income, jobs and houses should trigger additional remittance inflows from family members or relatives working abroad.^6^ In a separate specification, we control for this effect using data from the International Disaster Database.^7^ In particular, we retrieve all disasters in our sample countries with their exact dates and aggregate the number of affected people to a quarterly time series. We then include this series as well as six lags of it in our vector of control variables. Figure 4 reports the estimated coefficients. The response of remittances to monetary policy shocks remains virtually unchanged compared to the baseline result. Hence, our results are not affected by natural disasters driving remittances.

**Fig. 5:**
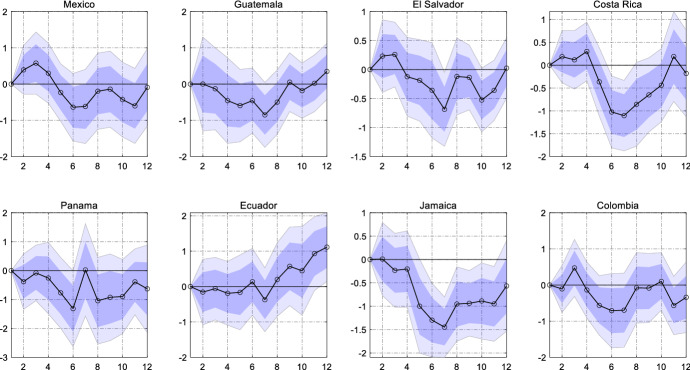
Country-specific response of remittance inflows to a US monetary policy shock. *Notes*: The graph shows the estimated country-specific $\beta_{ih}$ coefficients (black line). The dark (light) blue areas reflect 68% and 90% confidence intervals obtained from Driscoll and Kraay (1998) standard errors

In the model discussed before, we impose the restriction of equal slope coefficients across countries. The drawback of this assumption is that we cannot learn about the country-specific responses. In order to shed light on the potential cross-country heterogeneity, we re-estimate the model but allow country-specific slope coefficients ${\beta }_{ih}^{mon}$ for country *i*. As a result, we obtain eight impulse responses, one for each country, which are shown in Fig. 5.

After the tightening in the U.S., remittance inflows fall in all sample countries. Furthermore, the magnitude of the responses is not too different across countries. Remittances fall between 0.5% and 1%, with some responses (e.g. El Salvador, Costa Rica, Jamaica) exhibiting highly significant estimates, while the results for Mexico, Guatemala, Panama and Colombia are significant on the 68% level only. Inflows to Ecuador do not respond significantly. Overall, the results support the constraint of an equal slope coefficient for all countries in the panel.

In another specification, we re-estimate the model but separate positive from negative shocks. Hence, we split the Bu et al. (2021) shock into two depending on the sign of the shock: a tightening shock and an easing shock. The literature (see Tenreyro and Thwaites, 2016) shows that shocks of opposite signs could have different absolute effects on macroeconomic variables. The estimated model is

2 $$y_{i,t + h} = \alpha_{i,h} + \beta_{h}^{ + } \varepsilon_{t}^{mon, + } + \beta_{h}^{ - } \varepsilon_{t}^{mon, - } + \gamma_{h} {\varvec{X}}_{i,t} + \delta_{i,h} t + u_{i,t + h} ,$$

where ${\varepsilon }_{t}^{mon,+}$ and ${\varepsilon }_{t}^{mon,-}$ are tightening and easing shocks, respectively, and ${\beta }_{h}^{+}$ and ${\beta }_{h}^{-}$ are the associated coefficients. A fully symmetric response would imply ${\beta }_{h}^{+}{=\beta }_{h}^{-}$.

**Fig. 6:**
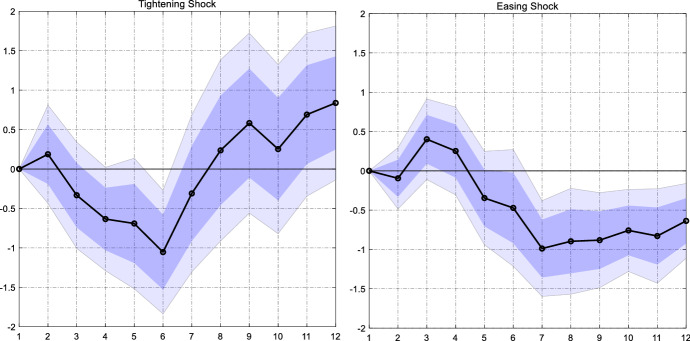
Response of remittance inflows to a US monetary policy shock when distinguishing between tightening and easing. *Notes*: The graph shows the estimated ${\beta }_{h}^{+}$ and ${\beta }_{h}^{-}$ from a model in which we separate tightening and easing shocks (black line). The dark (light) blue areas reflect 68% and 90% confidence intervals obtained from Driscoll and Kraay (1998) standard errors

The estimated coefficients of this specification are reported in Fig. 6. The coefficient on tightening shocks is significantly negative after six quarters. Hence, a tightening of one percentage point reduces inflows by about one percent. Likewise, the coefficient on the easing shock is also negative. If the Fed unexpectedly eases monetary conditions by one percentage point, inflows would increase by about one percent. Put differently, the coefficients are very similar and we do not find evidence for an asymmetric response of remittances. The only difference in the responses is the persistence: the effect of a tightening shock is short-lived, while remittances remain higher for up to 12 quarters after an accommodative monetary policy shock.

Figure 1 shows that for most sample countries FDI inflows are the second largest type source of funding. We now want to compare the sensitivity of remittance inflows to U.S. monetary policy with the responsiveness of FDI inflows to Fed policy. The response of FDI inflows is a useful benchmark to evaluate the economic significance of the effect of the Fed on the stream of remittances.

We draw net FDI inflows from the IMF International Financial Statistics. Unfortunately, other types of private capital inflows such that portfolio flows or bank flows are not available on a quarterly frequency for the full sample period. The dependent variable in the baseline model was the log of remittances in constant US dollar. In contrast to remittance inflows, FDI inflows can turn negative, such that we cannot apply the log operator. To scale the series, we express them as a share of domestic GDP. Since we ultimately want to compare the responses between remittances and FDI flows, we also-re-estimate the model with remittances expressed relative to GDP as the dependent variable. Finally, we standardize both dependent variables, remittances to GDP and FDI flows to GDP, such that we can compare the magnitudes of the estimated coefficients.

**Fig. 7:**
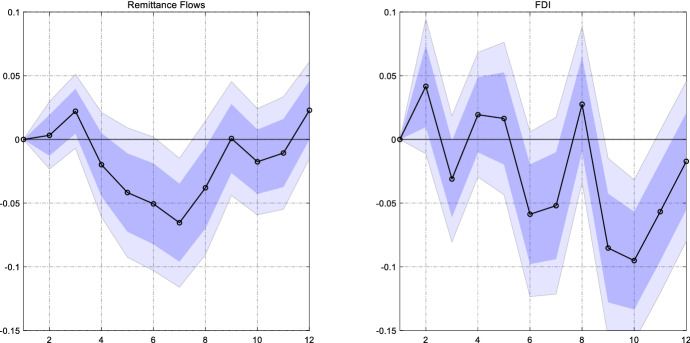
Comparing the responses of remittances and FDI flows to a US monetary policy shock**.** *Notes*: The graph shows the estimated ${\beta }_{h}$ for remittances as a share of GDP (left panel) and FDI inflows as a share of GDP (right panel). Both dependent variables are standardized. The dark (light) blue areas reflect 68% and 90% confidence intervals obtained from Driscoll and Kraay (1998) standard errors

Figure 7 plots the estimated coefficients for remittance flows and FDI flows. We find that the magnitude of the effect of the Fed on these types of flows is comparable, though the response of FDI flows tends to be a bit more volatile. This finding supports the quantitative importance of the remittance channel of monetary policy: the effect on remittances is as strong as the effect on FDI flows.

**Fig. 8:**
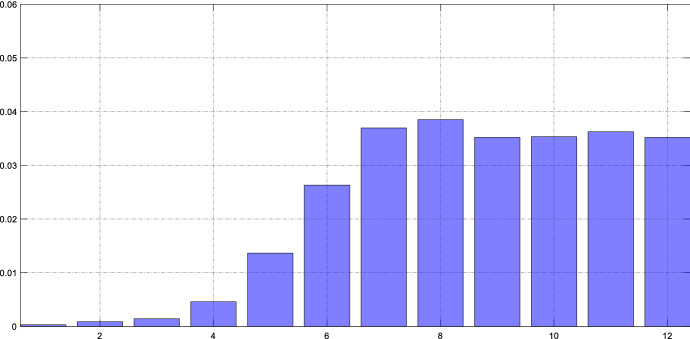
Contribution of US monetary policy shocks to remittance inflows. *Notes*: The bars show the share of unexpected fluctuations of remittance inflows at time *t* + *h* that is attributable to a US monetary policy shock occurring between *t* and *t* + *h*. This Forecast Error Variance Decomposition is obtained from the baseline model

The impulse responses reveal the effect of an unexpected marginal change in the stance of US monetary policy on remittance flows. In order to get an impression of the overall role of the Fed for remittances flows, we present the results not only in terms of impulse response functions, but also as forecast error variance decomposition. We follow Gorodnichenko and Lee (2020) and estimate the model without the monetary policy shock. Therefore, for each *h* we regress the residual on the shocks occurring between *t* and *t* + *h*. The *R*^2^ of this regression provides us with the share of fluctuations due to monetary policy. Figure 8 shows the fraction of unexpected fluctuations of remittance inflows that is explained by monetary policy shocks.

The numbers suggest that after one year monetary policy shocks explain between one and three percent of remittance fluctuations. In fact, the role of monetary policy is small relative to all other driving factors. This does not come as a surprise as shocks to income and employment other than monetary policy shocks explain the bulk of fluctuations of remittances. While the relative contribution of monetary policy as a driver of remittances remains small, we have established that the responses to policy shocks are statistically significant and economically relevant.

## The role of the exchange rate

It is well established that a policy tightening of the Fed results in a fall in income and employment in the US economy. We have shown that this contractionary effect also leads to a significant drop in remittance flows to emerging and developing countries in the Southern Hemisphere.

A key variable that deserves specific attention is the response of the real exchange rate to a Fed policy intervention. Standard models suggest that tighter monetary policy is the US should lead to a temporary real appreciation of the US economy. Everything else equal, the real appreciation should make it more attractive for migrants to send money to relatives back home. Hence, the US appreciation partly offsets the effect of lower income and employment on remittance flows.

**Fig. 9:**
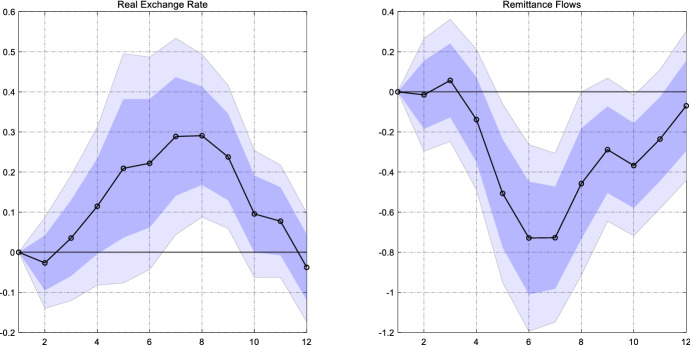
The response of the real exchange rate and remittance inflows to a US monetary policy shock. *Notes*: The graph shows the estimated $\beta_{h}$ for the real exchange rate relative to the US (left panel) and the inflows of remittances (right panel) with the real exchange rate as an additional control variable. The dark (light) blue areas reflect 68% and 90% confidence intervals obtained from Driscoll and Kraay (1998) standard errors

To shed light on the response of the exchange rate, we construct bilateral real exchange rate for each sample country using data from the IMF International Financial Statistics. The left panel of Fig. 9 shows the response of the real exchange rate to the policy shock emanating from the US. We spot a significant real depreciation by 0.3% after eight quarters. Hence, the real exchange rate responds as expected.

We then include the real exchange rate as an additional control variable in our baseline model specification in order to see whether the significant drop in remittances remains visible once we control for the exchange rate. The right panel of Fig. 9 reports the estimated coefficients. We still find a strong and significant fall in remittances by about 0.8% after six quarters. Put differently, or results survive once we take the exchange rate response into account.

## Conclusions

Remittance flows respond to economic developments in both the home country and the host country of migrant workers. In this paper, we address one specific driver of remittances to economies in Latin America and the Caribbean: the monetary policy of the US Federal Reserve. Our key result is that a monetary tightening significantly reduces remittance inflows. The results contribute to the understanding of the cross-border transmission of monetary policy and shed light on monetary conditions as a driver of remittance inflows, which has not yet been studied in the literature. As a matter of fact, the remittance-channel of monetary policy is only one channel through which the Fed affects countries in Latin America and the Caribbean. Among them are the Fed’s impact on the demand for exports from Latin America and the Caribbean, the value of the exchange rate or the reallocation of other forms of private capital flows.

One limitation of the current study is that we cannot distinguish between the extensive and the intensive margin. After a policy tightening and, as a consequence, a contraction in the US, migrant workers have less income available to send home. This is the intensive margin. In addition, the contraction of the US economy attracts fewer migrants from Latin America, this resulting in a smaller flow of remittances. This is the extensive margin. We leave an empirical analysis of this distinction for future research.

## Footnotes

1 See Chami et al. (2008) for a survey on the properties, determinants and consequences of remittance flows.

2 The impact of remittances on long-run economic growth is disputed, see Gapen et al. (2009).

3 The series is available at https://www.federalreserve.gov/econres/feds/a-unified-measure-of-fed-monetary-policy-shocks.htm.

4 Existing research using local projections (Bartscher et al., 2021; Bennani, 2021) shows that U.S. monetary policy has an unequal effect on unemployment of white, black or Hispanic workers. Ritter and Taylor (2011) and Hoynes et al. (2012) study the different effects of recessions for white, black and Hispanic workers.

5 See Higgins and Klitgaard (2020) and Kpodar et al. (2021) for an analysis of the behavior of remittance flows during the COVID-19 pandemic.

6 Bettin and Zazzaro (2018) suggest that remittances facilitate the process of reconstruction after natural disasters.

7 See https://www.emdat.be/.
